# Noncirrhotic Portal Vein Thrombosis Presenting with Hepatic Encephalopathy Due to Polycythemia Vera and MRI Findings

**Published:** 2017-04

**Authors:** Burcu GÖKÇE ÇOKAL, Tahir KURTULUŞ YOLDAŞ, Selda KESKİN GÜLER, Murat KEKİLLİ, Faruk PİRİNÇCİOĞLU, Hafize NALAN GÜNEŞ

**Affiliations:** 1. Dept. of Neurology, Ankara Education and Research Hospital, Ankara, Turkey; 2. Dept. of Gastroenterology, Ankara Education and Research Hospital, Ankara, Turkey

## Dear Editor-in-Chief

Hepatic encephalopathy (HE) is a neuropsychiatric syndrome commonly occurs with cirrhosis, portal hypertension, portal-systemic shunts and acute liver failure. The clinical features of HE vary between mild cognitive deficits to severe coma (1). Portal vein thrombosis (PVT) is a disorder which occurs by obstruction of the extra-hepatic portal vein with or without involvement of the intrahepatic portal veins (2). “The etiology of PVT can be local risk factors such as cirrhosis, hepatobiliary malignancies and pancreatitis, and systemic risk factors such as inherited and acquired prothrombotic disorders”(3). PVT is rarely seen in prothrombotic disorders in the absence of cirrhosis and the coincidence of hepatic encephalopathy is uncommon (3).

Here we describe the clinical presentation and brain magnetic resonance imaging (MRI) findings of a patient with HE and discovery of PVT, the etiology of probably attributed to polycythemia vera with Janus kinase 2 (JAK2) gene mutation.

**Fig. 1:**
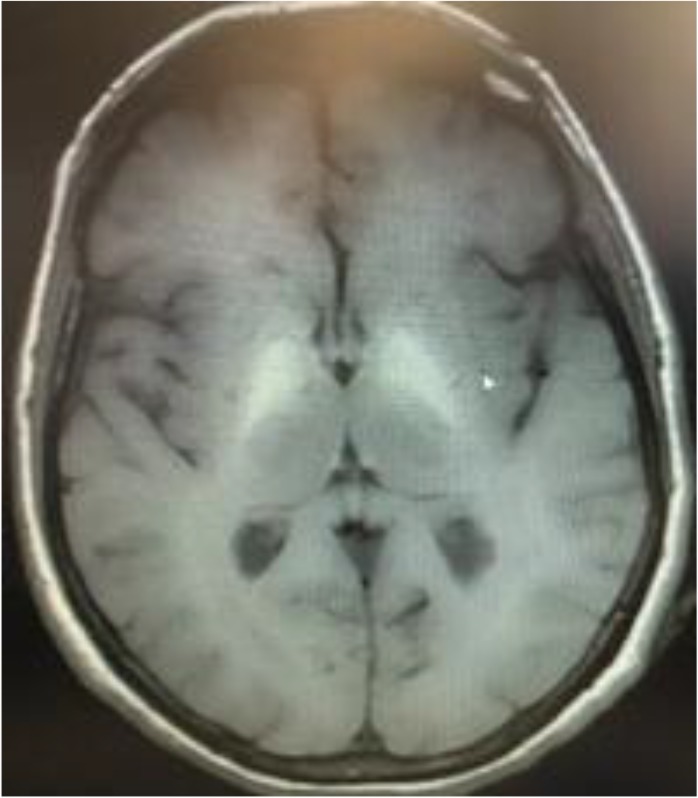
Bilateral and symmetrical T1 signal hyperintensity of the basal ganglia

**Fig. 2:**
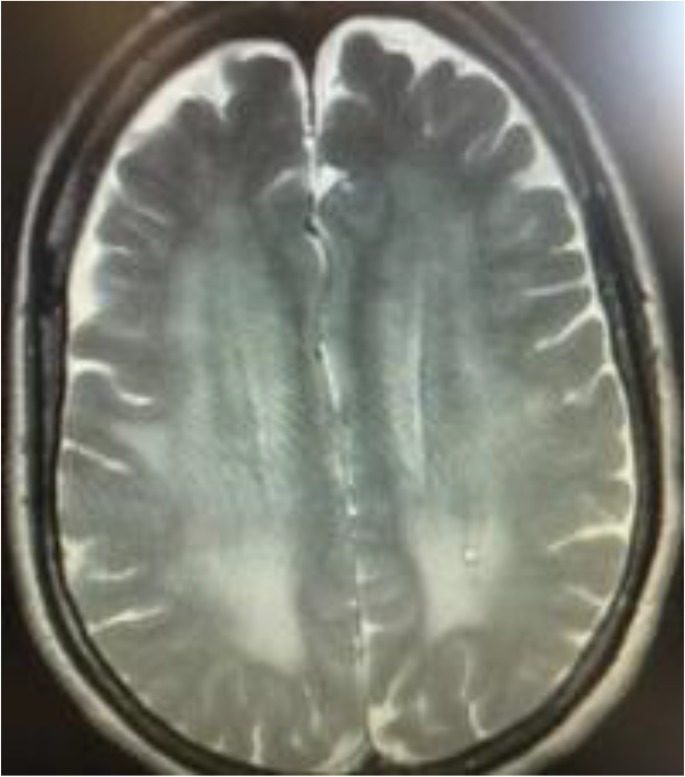
Diffuse hemispheric signal hyperintensity in white matter along the corticospinal tract on axial T2-weighted brain MRI sequence

A female patient 59 yr old was admitted for progressive mental alteration over a 24 h period. She was unconsciousness on admission. On neurological examination, she was non-cooperative, disoriented and lethargic. There was no focal neurological deficiency. Brain computerized tomography (CT) performed in the emergency room was normal. She was hospitalized in the intensive care unit. Routine laboratory findings were normal but the initial blood ammonia was elevated to 130 mmol/L (0 to 47 mmol/L as normal). After hospitalization, she has started antiaggregant 100 mg once daily. Brain MRI and electroencephalogram (EEG) were performed, immediately. The brain MRI revealed, bilateral and symmetrical T1 signal hyperintensity of the basal ganglia (Fig. 1) associated with diffuse hemispheric signal hyperintensity in white matter along the corticospinal tract on T2-weighted sequences (Fig. 2). There was dilation of the subarachnoid space around the optic nerve and flattening of the posterior aspect of the sclera that reflects increased intracranial pressure. There was no finding that indicates intracranial vascular events and intracranial space occupying lesions in the MRI of the patient. A diffuse theta slow wave pattern was seen on EEG. Diagnosis of probable HE was made. The patient was consulted to gastroenterology clinic and abdominal ultrasonography (USG) was performed. Abdominal USG revealed portal vein wall thickness and luminal narrowing and splenomegaly. HE diagnosis was confirmed by the gastroenterology clinic and the HE developed due to PVT. She has started the treatment of HE and her level of consciousness recovered on the second day. uring the follow-up her complete blood test showed marked thrombocytosis. Her platelet count was 900000 μL. A hematological consultation was requested and hematology department advised testing for JAK-2 gene mutation. The result of the test for JAK2-V617F gene mutation was positive, suggesting the presence of polycythemia vera.

Meanwhile, she recovered neurologically and she was discharged on anticoagulants with a target INR of 2–2.5 and advised to refer to the gastroenterology, neurology and hematology clinics for regular follow-up.

In HE portal blood directly shunts into systemic circulation bypassing liver and severe hepatocellular damage plays a role in pathogenesis of HE. Therefore, toxins not metabolized by liver and accumulate in the brain. The major toxins are known as ammonia, mercaptans, phenols and manganese (4). Ammonia crosses the blood– brain barrier and is absorbed and metabolized by the astrocytes. Increase in ammonia causes swelling of astrocytes and disturbs the blood-brain barrier permeability to various toxins which causes brain edema and increases intracranial pressure (ICP). The T2 hyperintensity seen on T2-weighted images is a common finding in patients with HE. The lesions can be observed in the cortex or in a diffuse pattern in white matter along the corticospinal tract caused by increased ammonia concentration (5).

Manganese (a paramagnetic substance) is also known to be responsible for bilateral symmetrical T1 hyperintensity in basal ganglia in HE patients. This finding has been reported in occupational exposures, liver cirrhosis and total parenteral nutrition with unbalanced solutions and in those with noncirrhotic portal vein thrombosis. Paramagnetic substances show enterohepatic circulation in portal vein thrombosis and cause the development of bright basal ganglia in T1 weighted MRI (6). In our patient, there was bilateral and symmetrical T1 signal hyperintensity of the basal ganglia on MRI, which shows the manganese deposits and diffuses hemispheric signal hyperintensity in white matter along the corticospinal tract on T2-weighted sequences, which suggest hyperammonemia. Dilation of the subarachnoid space around the optic nerve and flattening of the posterior aspect of the sclera also reflects increased intracranial pressure probably due to brain edema associated with hyperammonemia. Although PVT associated with HE is a rare condition clinician, it should consider the possibility of HE and PVT while evaluating comatose patients. When PVT diagnosis is made in the absence of cirrhosis, inherited or acquired prothrombotic disorders and myeloproliferative diseases should always be considered.
